# Steam Explosion-Assisted Extraction of Polysaccharides from *Pleurotus eryngii* and Its Influence on Structural Characteristics and Antioxidant Activity

**DOI:** 10.3390/foods13081229

**Published:** 2024-04-17

**Authors:** Jianqing Qiu, Peiying Zheng, Wanzhen Dai, Zhijun Zheng, Xiaohui Lin, Jiamiao Hu, Shaoxiao Zeng, Shaoling Lin

**Affiliations:** 1 College of Food Science, Fujian Agriculture and Forestry University, Fuzhou 350002, China; qjqqiu@163.com (J.Q.); zhengpy19990520@163.com (P.Z.); wanzhen.dai@fafu.edu.cn (W.D.); jh921@leicester.ac.uk (J.H.); zsxfst@163.com (S.Z.); 2 College of Food and Bioengineering, Fujian Polytechnic Normal University, Fuqing 350300, China; 3 Fujian Subtropical Fruit Beverage Engineering Research Center, Zhangzhou 363000, China; kzw@vip.163.com (Z.Z.); lxh8029@163.com (X.L.); 4 College of Life Sciences, University of Leicester, Leicester LE1 7RH, UK; 5 Integrated Scientific Research Base of Edible Fungi Processing and Comprehensive Utilization Technology, Ministry of Agriculture and Rural Affairs, Fuzhou 350002, China

**Keywords:** steam explosion, polysaccharide, *Pleurotus eryngii*, antioxidant capacity

## Abstract

*Pleurotus eryngii* (PE) has been sought after for its various health benefits and high content of phenolic compounds. This study explored the feasibility of steam explosion (SE)-assisted extraction of polysaccharides with high antioxidant capacities from PE. An orthogonal experimental design (OED) was used to optimize the SE-assisted extraction of PE. The influence of the optimized SE-assisted extraction on the physicochemical properties of PE polysaccharides was determined by scanning electron microscopy (SEM), Fourier transform infrared spectroscopy (FTIR), monosaccharide compositional analysis and antioxidant capacity assays. Under optimal SE conditions, SE-assisted extraction increased the polysaccharide yield by 138% compared to extraction without SE-assistance. In addition, SEM demonstrated that SE-assisted extraction markedly altered the spatial structure of *Pleurotus eryngii* polysaccharides (PEP), and monosaccharide compositional analysis revealed that this pretreatment significantly increased the proportions of some monosaccharides, such as glucose, rhamnose and arabinose, in the isolated PEP. FTIR spectra indicated no change in the major chemical functional groups of PEP. PEP extracted by SE-assisted extraction had significantly increased free radical scavenging and antioxidant capacities. In conclusion, SE-assisted extraction appears to be a novel polysaccharide extraction technology, which markedly increases extraction yields and efficiency and can increase the biological activity of polysaccharide extracts.

## 1. Introduction

*Pleurotus eryngii* (PE), also known as the king trumpet mushroom or king oyster mushroom [1], is a commercially important species of edible mushroom. PE is well known for its high nutritional value not just because of its relatively high content of protein, fiber, minerals, unsaturated fatty acids, trace elements and secondary metabolites but also because of its low fat content, [2,3]. PE mushrooms are valued for their desirable taste, low fat content and traditional Chinese medicinal applications, which result in a high commercial value [4]. PE is rich in biologically active substances, such as polysaccharides, dietary fiber, lipids, peptides and sterols [5], which confer a variety of pharmacological activities [6]. One of the most extensively studied components is *Pleurotus eryngii* polysaccharides (PEP). Numerous studies have shown that PEP has multiple health-promoting functions, such as antioxidant, anti-tumor, improving immunity, hepatoprotection, and anti-hyperlipidemia [7]. Efficient extraction of polysaccharide from PE and exploration of its structural properties and biological activity is the subject of increasing research interest [2,8,9], which will facilitate its more extensive application in the functional food and pharmaceutical industries [10].

In recent years, various methods have been developed to extract bioactive polysaccharides, including physicochemical (SE-assisted extraction, hydrothermal, fiber expansion with ammonia), chemical (dilute acids, alkalis, ionic liquids, enzymatic, oxidizing agents, organic solvents), physical (grinding, irradiation, extrusion) and biological (fermentation) [11,12] techniques. Hot water extraction is the most commonly used of these extraction methods, with the advantages of mild conditions and simple operation, but this method often results in low polysaccharide yield, high energy consumption and low biological activity of the extracted polysaccharide [13]. The alkaline extraction method improves the polysaccharide yield and biological activity and is suitable for the extraction of acidic polysaccharides (i.e., those containing glucuronic acid), but the usage of alkali may degrade the polysaccharide structure and it modifies its physicochemical and biological properties [14]. Currently, ultrasonic extraction has been developed in combination with chemical extraction methods, which can reduce interference by solvents and improve the polysaccharide yield, but precise control of the ultrasonic power and extraction time is required to prevent undesirable changes in molecular structure [15]. The enzyme-assisted extraction method is also suitable for the extraction of plant polysaccharides with low polysaccharide content or those that are affected by solvents and prone to structural changes. However, enzymes are relatively expensive, and their biological activities can be affected by a variety of factors, such as enzyme concentration, temperature, time and pH [16]. The supercritical fluid extraction method is also reported to effectively extract polysaccharides, but the required equipment is complex and the operating cost is high [17].

SE-assisted extraction has been used to extract bioactive components with relatively high yields, and it is a novel thermal processing technology with high efficiency and low energy consumption, which has advantages such as simple pretreatment, short processing time, freedom from chemical residues and low cost [18]. SE-assisted extraction is widely applied to improve the extraction yields of intracellular water-soluble bioactive components such as polysaccharides [11], phenols [10,19], dietary fiber [20,21] and flavonoids [22,23]. Physical treatments are generally the most efficient, since they are rapid, easy to use, inexpensive and leave no chemical residues. The physical, chemical and functional properties of polysaccharides are largely determined by their macromolecular structure, molecular weight, monosaccharide composition and functional groups.

SE-assisted extraction is a hydrothermal treatment, involving high pressure steam [24], which is different from most other thermal treatment processes. The rapid pressure release results in a major disruption of plant or fungal material, enabling efficient extraction. The material to be extracted is placed into a pressure chamber and treated with steam at high temperature and pressure, followed by instantaneous pressure release, which causes an explosive expansion of the steam permeating the material, resulting in cell wall and tissue disruption [11]. Various chemical reactions are promoted by the prolonged residence time in the high pressure steam [25]. Tissue disruption facilitates the subsequent extraction of polysaccharides and other components, increasing extraction yields and potentially modifying their biological activities [26].

After SE-assisted extraction, the extraction yield of flavonoids from sumac fruit was almost eight times higher than that of a non-SE-assisted extraction [27]. Phenolic compounds bound to polysaccharides in the cell wall have also been successfully released by steam explosion. SE-assisted extraction of grape pomace increased both the extraction yield of phenolic compounds and their antioxidant capacity [20]. SE-assisted extraction disrupted the cellular structure of soybean seed coats, improving both the extraction efficiency of the aglycone and acetyl-glucoside forms of isoflavonoids and their antioxidant capacity [18]. SE-assisted extraction can modify the polyphenolic composition and increase the cellular antioxidant and anti-proliferative activities of *Adinandra nitida* leaves [28]. SE-assisted extraction improves the extraction yield of polysaccharides while altering their structural characteristics and biological activities [10,29,30]. The effects of SE-assisted extraction on plant tissue include thermal degradation, mechanical decomposition, hydrogen bond disruption and acid hydrolysis [10].

Although SE pretreatment has shown a number of advantages in polysaccharides extraction, to the best of our knowledge its application in the extraction of PE polysaccharides remains unexplored. The main concern in polysaccharide applications is how to modify their structure and physicochemical properties while optimizing the extraction conditions and increasing the extraction yield. Accordingly, the aim of this study was to explore the potential of efficiently extracting PE polysaccharides with high antioxidant capacity using SE-assisted extraction. The impacts of explosion temperature, pressure, time and other factors on the yield of polysaccharides was determined, as well as the effects of SE-assisted extraction on the structural characteristics of PE polysaccharide (PEP) extracts. Furthermore, the effect of SE-assisted extraction on the antioxidant capacity of the polysaccharide extracts was determined. The antioxidant capacities of the SE-assisted extract of *Pleurotus eryngii* polysaccharide (SEPEP) and PEP were also compared.

## 2. Materials and Methods

### 2.1. Reagents and Plant Material

Fresh *Pleurotus eryngii* (PE) was purchased from a local supermarket (Fujian, China). The monosaccharide standards were obtained from the National Institutes for Food and Drug Control (Beijing, China). The antioxidant assay kits (including 2,2′-azinobis (3-ethylbenzthiazoline-6-sulfonic acid (ABTS) method kit, 1,1 diphenyl-2-picrylhydrazyl (DPPH) method kit and hydroxyl radical (**^·^**OH) method kit) were obtained from Beijing Solarbio Science and Technology Co., Ltd. (Beijing, China). Other chemicals and reagents used were obtained from local distributors and were analytical grade.

### 2.2. Steam Explosion (SE)-Assisted Extraction

**Figure 1:**
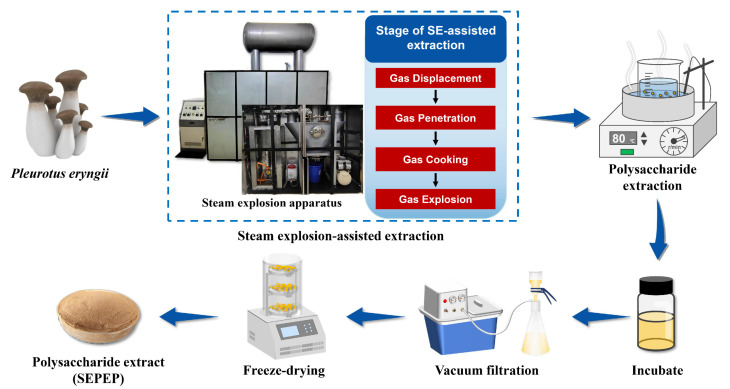
Schematic diagram of SEPEP extraction with steam explosion pretreatment.

The SE-assisted extract of *Pleurotus eryngii* polysaccharide (SEPEP) was prepared using a steam explosion apparatus (QB-200, Qingzheng Eco-Technology (Suzhou) Co., Ltd., Suzhou, China). The scheme of SEPEP extraction with steam explosion pretreatment is illustrated in Figure 1.

Approximately 100 g of dried PE, rehydrated to a water content of 20–30%, was placed into the SE chamber and treated at steam pressures from 0.8–1.6 MPa for 30–90 s with saturated steam. The set temperature was reached within a few seconds, then a ball valve at the bottom of the reactor was opened to rapidly release the pressure inside the reactor, subjecting the PE material to a steam explosion. The resulting slurry of disrupted PE material was collected in the receiver [31]. The SE-extracted PE samples were collected and dried at 60 °C until the water content decreased to 10%, then ground and sieved with an 80-mesh screen. PE powder samples were extracted under various conditions after dispersion in distilled water, at temperatures from 50–90 °C, for times from 1–5 h and at solid/liquid ratios from 1:10–1:50 g/mL. Each extract was filtered through a 200-mesh screen, after which the filtrate was concentrated to 1/10 of the original sample volume via rotary vacuum evaporation. The concentrate was suspended in a 4-fold volume of 95% ethanol and then stored at 4 °C for 24 h. The resulting precipitate was separated by vacuum filtration, then freeze-dried to obtain crude PEP. Statistical optimization of the process parameters for the SE-assisted extraction of PEP was performed using a Taguchi orthogonal array design; the ranges and variables are shown in Table 1. Based on this design, an orthogonal array of 8 experiments (L_8_) was formulated to optimize the process parameters for SE-assisted extraction (Table 2). Analysis of variance (ANOVA) was performed to identify the statistically significant factors. The extraction yield of PEP was determined using the following equation:

(1) $$Extraction\;yield\left(\%\right)=m_{2}{/m}_{1}\times 100\%$$

where m_1_ represents the weight of PE and m_2_ represents the weight of PEP.

### 2.3. Scanning Electron Microscopic (SEM) Observation

The surface morphologies of PEP and SEPEP were observed using an SEM (Zeiss Merlin Compact, Jena, Germany). The samples were coated with a thin gold layer and placed on the substrate, and images were recorded at 1.0 kV at 500×, 1000× and 2000× magnification, at Sanshu Biotech. Co., LTD (Shanghai, China).

### 2.4. Fourier Transform Infrared Spectroscopy (FTIR) Analysis

FTIR spectra of polysaccharides were recorded using a Nicolet iZ-10 spectrometer (Thermo Nicolet, Minneapolis, MN, USA). The polysaccharide samples were mixed with KBr powder and then pressed into 1 mm pellets for FTIR measurement in the range of 4000–400 cm^−1^ at Sanshu Biotech.

### 2.5. Determination of Monosaccharide Composition

The monosaccharide composition was determined as described previously, with minor modifications [32]. The PEP sample was hydrolyzed with 2 mol/L trifluoroacetic acid at 110 °C for 4 h and cooled to room temperature. The solution was then added to methanol (1 mL), purged with nitrogen at 40 °C until dry, then dissolved in deionized water (0.5 mL). An aliquot of the aqueous solution (0.1 mL) was added to a sodium hydroxide solution (0.1 mL, 0.3 mol/L) and the hydrolyzed products were derivatized by methanolic 1-phenyl-3-methyl-5-pyrazolone (PMP, 0.1 mL, 0.5 mol/L), then heated at 70 °C for 60 min. After cooling, the sample was neutralized with hydrochloric acid (0.1 mL, 0.3 mol/L), then centrifuged at 5000× *g*, then the supernatant was extracted with dichloromethane (3 × 1 mL). The aqueous layer was filtered through a 0.22 μm polyethersulphone membrane, then analyzed by HPLC (Waters E2695, Waters, Milford, MA, USA), The derivatized products were analyzed on a Waters SunFire C18 column (4.6 × 250 mm). Authentic standards of mannose (Man), rhamnose (Rha), glucuronic acid (GlcA), galacturonic acid (GalA), glucose (Glc), galactose (Gal), xylose (Xyl), arabinose (Ara) and fucose (Fuc) were also derivatized using the same method.

### 2.6. DPPH Free Radical Scavenging Assay

A slightly modified version of a previously described method [33] was used to determine the DPPH radical scavenging capacity of the PE extract, using a DPPH free radical assay kit (Beijing Solarbio Science and Technology Co., Ltd., Beijing, China). DPPH (1,1-diphenyl-2-picrylhydrazyl radical) is a stable nitrogen free radical [31]. The free radical scavenging capacity was determined from the change in absorbance resulting from the reduction of DPPH relative to that of the control [34]. PE extract (1 mg/mL, 25 μL) from SE-assisted extraction and a freshly prepared DPPH ethanol solution (156 μL) were added to ethanol (819 μL) and mixed. The mixtures were centrifuged (2500× *g*, 10 min) after 30 min of reaction (room temperature, in the dark), then the DPPH scavenging capacity was determined by measuring the absorbance at 515 nm using spectrophotometry (UV756, Yoke instruments, Shanghai, China) with ascorbic acid as a positive control. Each sample was measured in triplicate and the mean and standard deviation (*n* = 3) were calculated. The free radical scavenging capacity was calculated using the following formula:

(2) $$DPPH\;free\;radical\;scavenging\;capacity\left(\%\right)=[A_{0}-{(A}_{1}-A_{2})/A_{0}]\times 100\%$$

where A_0_ is the absorbance of sample and DPPH ethanol solution, A_1_ is the absorbance of supernatant and DPPH ethanol solution and A_2_ is the absorbance of supernatant and ethanol.

### 2.7. ABTS Free Radical Scavenging Assay

A slightly modified version of a previously described method [35] was used to determine the ABTS radical scavenging capacity of the PE extract using an ABTS free radical scavenging assay kit (Beijing Solarbio Science and Technology, Beijing, China). Stable blue-green cationic 2,2′-azino-bis (3-ethylbenzothiazoline-6-sulfonate) (ABTS) free radicals are generated after the oxidation of ABTS. The antioxidant components in the sample scavenged the ABTS free radicals and reduced the color intensity. Briefly, SE-assisted PE extract (50 μL, 1 mg/mL) was mixed with ABTS solution (950 μL) and the mixture was reacted for 6 min (room temperature, in the dark). The ABTS scavenging capacity was determined at 405 nm by spectrophotometry (UV756, Yoke instruments, Shanghai, China) with ascorbic acid as a positive control. Each sample was measured in triplicate and the mean and standard deviation (*n* = 3) were calculated.

The ABTS free radical scavenging capacity was calculated with the following formula:

(3) $$Free\;radical\;scavenging\;capacity\left(\%\right)=[A_{0}-{(A}_{1}-A_{2})/A_{0}]\times 100\%$$

where A_0_ is the absorbance of distilled water and ABTS solution, A_1_ is the absorbance of supernatant and ABTS solution, and A_2_ is the absorbance of supernatant and distilled water.

### 2.8. Hydroxyl Radical Scavenging Capacity Assay

A slightly modified version of a previously reported method [36] was used to determine the hydroxyl radical scavenging capacity of the PE extract using a hydroxyl radical scavenging assay kit (Beijing Solarbio Science and Technology, Beijing, China). The assay is based on the interaction between hydrogen peroxide and iron; the hydrogen peroxide oxidizes Fe^2+^ in the O-diazophenanthrene complex indicator to Fe^3+^, resulting in a decrease in absorbance at 536 nm. The degree of inhibition of the rate of absorbance decrease reflects the capacity of the antioxidants present to scavenge hydroxyl radicals. SE-assisted PE extract (1 mg/mL) was mixed with 9 volumes of assay solution by vortexing, then placed in a water bath at 37 °C for 60 min. The solution was then centrifuged at 5000× *g* for 10 min at room temperature. The hydroxyl radical scavenging capacity was determined from the absorbance change at 405 nm observed using spectrophotometry (UV756, Yoke instruments, Shanghai, China). Each sample was measured in triplicate and the mean and standard deviation (*n* = 3) were calculated.

The hydroxyl radical scavenging capacity was calculated with the following formula:

(4) $$Free\;radical\;scavenging\;capacity\left(\%\right)=(A_{2}-A_{1}){/(A}_{0}-A_{1})\times 100\%$$

where A_0_ is the absorbance of the reagent blank, A_1_ is the absorbance of the control and A_2_ is the absorbance of the sample.

### 2.9. Statistical Analysis

Results are expressed as the mean ± standard deviation (SD) of three replicates. Statistical analyses were performed using SPSS 26.0 software (SPSS, Inc., Chicago, IL, USA). Selected experimental data were analyzed using GraphPad Prism 8.0.0 by one-way analysis of variance (ANOVA). Data were subjected to Duncan’s post hoc test. Differences were considered statistically significant at * *p* < 0.05, ** *p* < 0.01, *** *p* < 0.001 and **** *p* < 0.0001. The figures were constructed using Origin 2022 (Origin Lab, Northampton, MA, USA).

## 3. Results and Discussion

### 3.1. Determination of Optimal Steam Explosion (SE)-Assisted Extraction Conditions

**Figure 2:**
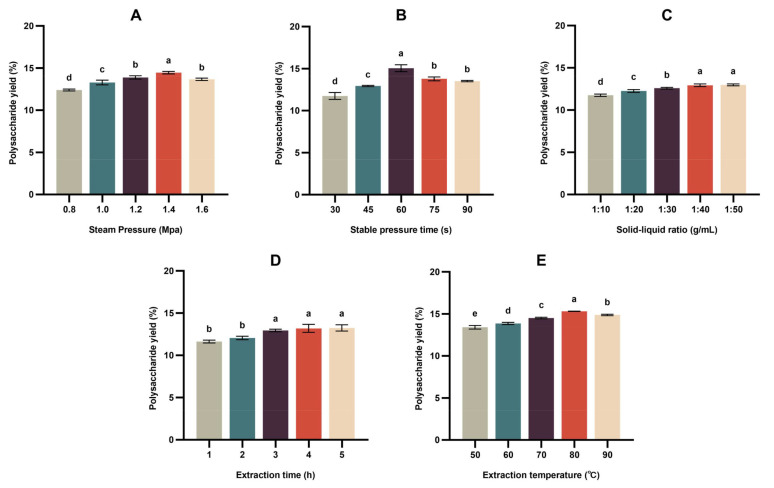
Optimization of steam explosion (SE)-assisted extraction process parameters, steam pressure (**A**), stabilization time (**B**) and solid-liquid ratio (**C**), extraction time (**D**) and extraction temperature (**E**). Different letters mean results differ significantly at *p* < 0.05.

The SE-assisted extraction variables, steam pressure, stabilization time and solid/liquid ratio were varied separately (Figure 2) to assist in the selection of values for the Taguchi experimental design. Varying the extraction pressure (Figure 2A) increased the PEP yield gradually to a maximum at 1.4 MPa, then decreased it at 1.6 MPa. Varying the stabilization time (Figure 2B) produced a similar result, with a clear maximum PEP yield at 60 s. The solid-liquid ratio (Figure 2C) had little effect on the PEP yield, with equal maxima at 1:4 and 1:5. Extraction time (Figure 2D) behaved similarly, with equal maxima at 4 and 5 h. Varying the extraction temperature (Figure 2E) increased the PEP yield gradually to a maximum at 80 °C.

It appears that at the highest values of extraction temperature, extraction pressure and stabilization time, the degradation of polysaccharides is maximized and outweighs the increase in extraction efficiency, thereby lowering the PEP yield [37].

The results of the above single-factor optimization were used to aid the formulation of the Taguchi experimental design. To optimize the process parameters and maximize the PEP yield, the optimal SE-assisted extraction conditions were found to be 1.2 MPa steam pressure, 60 s stabilization time, 1:40 g/mL solid-liquid ratio and treatment at 80 °C for 4 h (Table 3). R-value comparisons indicate that the relative influence of the process parameters was solid-liquid ratio > steam pressure > stabilization time > extraction time > extraction temperature. The PEP extraction was carried out under the above optimized conditions, both without (PEP) and with (SEPEP) SE-assisted extraction; the polysaccharide yield significantly increased (*p* < 0.05) from 7.52 ± 0.08 to 17.89 ± 0.40%, respectively (Table 4). Previous studies of PEP extraction have reported that ultrasonic extraction reached a 7.5 ± 0.3% yield of PEP [38], while extraction of PE powder with ethanol as the solvent showed a 7.31 ± 0.15% yield [39] and alkaline extraction demonstrated a 6.3% yield [40]. These results were lower than the PEP yields obtained during this study, showing the beneficial effects of SE-assisted extraction on the increased yields.

### 3.2. Effects of Steam Explosion (SE)-Assisted Extraction on the Microstructure of Pleurotus eryngii Polysaccharides

**Figure 3:**
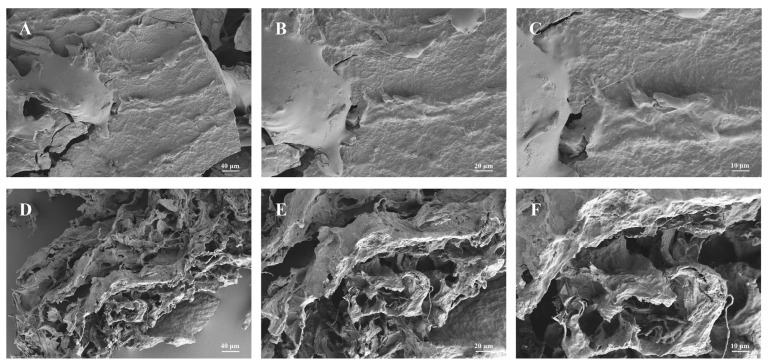
Surface morphologies of PEP ((**A**) 500×; (**B**) 1000×; (**C**) 2000×) and SEPEP ((**D**) 500×; (**E**) 1000×; (**F**) 2000×)), observed by scanning electron microscopy.

The surface morphologies of PEP and SEPEP are shown in Figure 3, at magnifying powers of 500×, 1000× and 2000×. The untreated PEP appeared as blocky aggregates with a smooth compact surface and minimal visible fragmentation, indicating the presence of strong intermolecular interactions and the formation of a tight structure. After SE-assisted extraction, the surface morphology of PEP changed markedly. The surface irregularity markedly increased and the structure became sponge-like, indicating that steam explosion promotes the formation of new cross-linkages between the partially-degraded polysaccharide molecules [10]. The high temperature, pressure and shear forces present during SE-assisted extraction may rearrange linkages between monosaccharide residues as well as breaking them [41], which could explain the changes in physicochemical properties. Indeed, previous studies have shown that steam blasting during SE pretreatment could destroy or disrupt tight cross-linking between polysaccharides and proteins and create a more porous structure with an increased surface area, resulting in enhanced dissolution of the constituent components and likely explaining the observed augmentation in PEP yield. This is in agreement with a previous report that SE-assisted extraction changed the surface of the native polysaccharide particles from smooth aggregated structures to a porous honeycomb structure [12].

### 3.3. Monosaccharide Compositional Analysis of Pleurotus eryngii Polysaccharides

**Figure 4:**
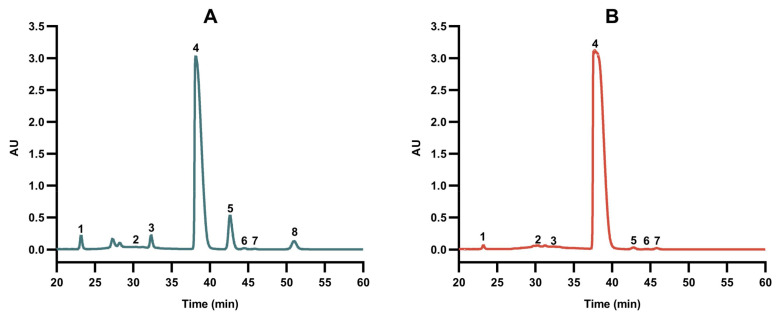
HPLC chromatograms of the monosaccharide composition of PEP (**A**) and SEPEP (**B**). Peaks: 1—mannose; 2—rhamnose; 3—glucuronic acid; 4—glucose; 5—galactose; 6—xylose; 7—arabinose; 8—fucose.

The monosaccharide composition of PEP was dominated by glucose, but mannose, rhamnose, galacturonic acid, galactose, xylose, arabinose and fucose were also present (Figure 4). SE-assisted extraction significantly increased the relative contents of glucose, rhamnose and arabinose, whereas those of the other monosaccharides markedly decreased, suggesting that the polysaccharide backbone may be mainly composed of glucose residues. The hydrolysis of insoluble cellulose into soluble cello-oligosaccharide fragments by SE-assisted extraction may have increased the glucose content of PEP [42]. SE-assisted extraction significantly decreased the content of mannose, galacturonic acid, galactose, xylose and fucose. This may be the result of high temperature and high pressure promoting the dehydration of mannose, galacturonic acid, galactose, xylose and fucose into products containing 5-furfural and 5-hydroxymethylfurfural [43].

### 3.4. FTIR Analysis of Pleurotus eryngii Polysaccharides (PEP)

**Figure 5:**
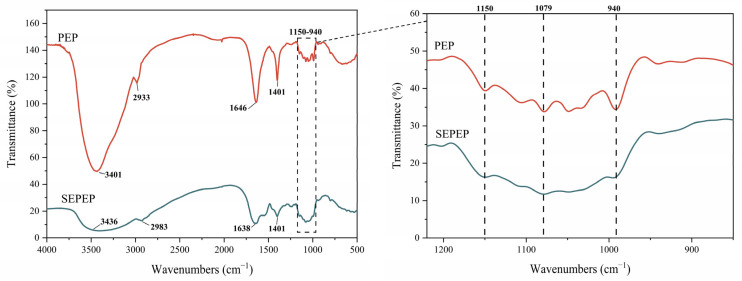
FTIR spectra of PEP and SEPEP, with an expansion of the 800–1250 cm^−1^ region.

The FTIR spectra of PEP and SEPEP showed the characteristic absorption peaks of polysaccharides (Figure 5). The strong broad absorption of PEP at 3401 cm^−1^ and SEPEP at 3436 cm^−1^ corresponds to the O-H group stretching vibration. The absorptions at 2933 cm^−1^ (PEP) and 2983 cm^−1^ (SEPEP) correspond to the C-H group stretching vibration, i.e., the C-H stretching vibrations of CH_2_ groups in free sugars [44]. The absorptions at 1646 cm^−1^ (PEP) and 1638 cm^−1^ (SEPEP) correspond to the C=O group stretching vibration [45]. The intense and broad bands at nearly 1401 cm^−1^ probably correspond to the C-H flexing vibration [46], which has a relatively low intensity [41]. The absorbances of PEP and SEPEP at 1150–940 cm^−1^ correspond to C-O-C stretching and C-OH bending, confirming the presence of pyranose rings. Therefore, the FTIR spectra indicate that the major functional groups of the polysaccharides were unaltered by SE-assisted extraction [12].

### 3.5. Effects of Steam Explosion (SE)-Assisted Extraction on the Antioxidant Capacity of Pleurotus eryngii Polysaccharides (PEP)

**Figure 6:**
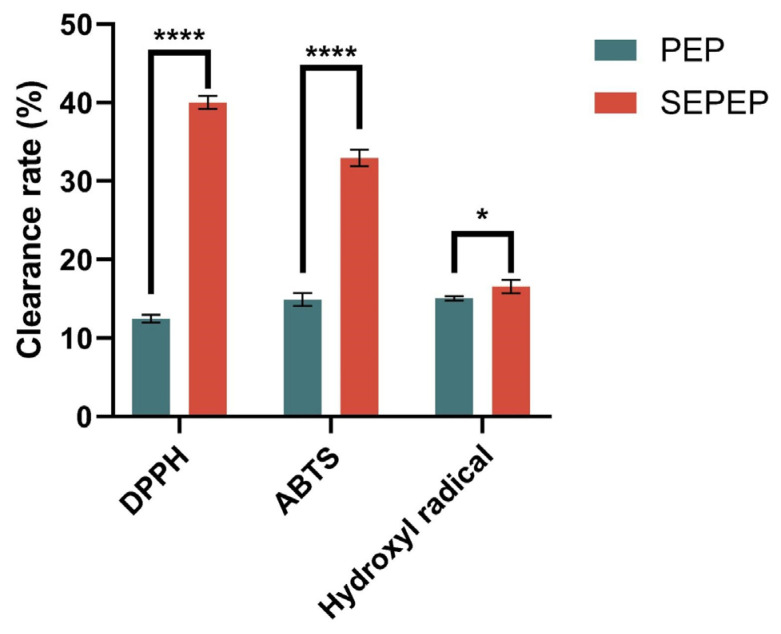
Free radical scavenging capacity of PEP and SEPEP. Each value is represented as the mean ± SD (*n* = 3). “*” means statistically significant differences at *p* < 0.05; “****” means statistically significant differences at *p* < 0.0001.

Plant polysaccharides are effective biological antioxidants, which help to remove harmful reactive oxygen species (ROS) and reduce cellular oxidative stress [30]. Since phytochemicals have a wide variety of structures, functional groups and combinations of groups, multiple antioxidant assays must be used to properly characterize their antioxidant activities. The DPPH, ABTS and hydroxyl radical scavenging capacity assays are widely accepted as being rapid and sensitive, so they were used to analyze the in vitro antioxidant capacity of PEP and SEPEP. The radical scavenging capacity of PEP and SEPEP was determined using the DPPH, ABTS and hydroxyl radical assays. Overall, SE-assisted extraction markedly increased the three free radical scavenging capacities of PEP (Figure 6; *p* < 0.05). The DPPH free radical scavenging capacity of PEP increased from 12.46 ± 0.47 to 40.02 ± 0.82%, the ABTS capacity from 14.89 ± 0.83 to 32.94 ± 1.05% and the hydroxyl radical capacity increased from 15.07 ± 0.31 to 16.55 ± 0.86%. Ultrasonic extraction of PEP [38] resulted in a polysaccharide DPPH free radical scavenging capacity of 16.8 ± 1.4% and a hydroxyl radical scavenging capacity of 16.1 ± 1.5%, which are lower than those of SE-assisted extraction. Therefore, SE-assisted extraction markedly increased the antioxidant capacity of PEP. This marked increase may result from the increase in the uronic acid content of SEPEP. A high uronic acid content in polysaccharides increases their free radical scavenging capacity [10]. Similarly, SE-assisted extraction of phytochemicals from Java tea enhanced the antioxidant capacity of the extracts [47], which is consistent with the findings of this study.

## 4. Conclusions

Taken together, the findings from this study demonstrated that steam explosion (SE)-assisted extraction significantly improved the efficiency of polysaccharide extraction from *Pleurotus eryngii* (PE) mushrooms. Under optimal treatment conditions, SE-assisted extraction increased the polysaccharide yield by 138% compared to extraction without SE pretreatment (17.89 ± 0.40 vs. 7.52 ± 0.08%).

Physicochemical analyses revealed that SE pretreatment markedly altered the microstructure of the extracted PEP particles, forming markedly increased surface irregularity and a sponge-like structure, whereas PEP without SE pretreatment had a smooth, blocky appearance. SE-assisted extraction also cleaved glycosidic linkages, apparently degrading the larger polysaccharides and modifying the compositions of their monosaccharides. In addition, FTIR spectral analysis indicated that the major functional groups of the polysaccharides were unaltered by SE-assisted extraction.

It has been suggested in previous reports that the physicochemical properties of polysaccharides, such as their solubility, monosaccharide composition, molecular weight, and the contents of positively or negatively charged groups, are determining factors for the magnitude of their different antioxidant actions. Therefore, the structural and chemical changes induced in PEP by SE-assisted extraction could contribute to an increased free radical scavenging capacity. This indicates that SE-assisted extraction could serve as an efficient, novel and environmentally friendly thermal processing technology, which can increase the polysaccharide extraction yield from PE and potentially other fungal and plant materials. In addition, SE-assisted extraction is able to enhance the antioxidant capacity of the extracted polysaccharides and potentially enhance other health-beneficial biological activities.

However, it should be noted that prolonged use of high temperatures and pressures can cause the degradation or oxidation of food components, especially polysaccharides. In addition, the application of SE-assisted extraction may degrade bioactive components or induce Maillard reactions between proteins and carbohydrates. Furthermore, a range of previous literatures have also suggested that SE-assisted extraction consistently leads to a reduction in the molecular weight of polysaccharides due to the breaking of internal glycosidic bonds induced by the steam blast [12,29] and may influence the branching structure of the polysaccharides [11,48]. These impacts may further affect the functionalities of polysaccharides. Therefore, further research is needed to investigate the overall impact of SE-assisted extraction on the nutritional value and safety of foods. For example, whether and how SE pretreatment affects the phenolic compounds covalently linked to polysaccharides should be further explored. SE has long been an important industrial process for physical biomass processing. SE-assisted extraction appears to have considerable potential for the extraction and chemical modification of bioactive food components in the food processing industry.

## Figures and Tables

**Table 1:** Factors and their respective levels for the Taguchi experimental design.

Factors	Levels	
	1	2
(A) Steam pressure (MPa)	1.2	1.4
(B) Stable pressure time (s)	60	75
(C) Solid-liquid ratio (g/mL)	1:40	1:50
(D) Extraction time (h)	3	4
(E) Extraction temperature (°C)	80	90

**Table 2:** Taguchi L_8_ (2^7^) orthogonal array design.

Runs	(A) Steam Pressure (MPa)	(B) Stable Pressure Time (s)	(C) Solid-Liquid Ratio (g/mL)	(D) Extraction Time (h)	(E) Extraction Temperature (°C)
1	1 (1.2)	1 (60)	1 (1:40)	1 (3)	1 (80)
2	1	1	1	2 (4)	2 (90)
3	1	2 (75)	2 (1:50)	1	1
4	1	2	2	2	2
5	2 (1.4)	1	2	1	2
6	2	1	2	2	1
7	2	2	1	1	2
8	2	2	1	2	1

**Table 3:** Taguchi L_8_ (2^7^) Orthogonal array design and experimental results.

Runs	(A) Steam Pressure (MPa)	(B) Stable Pressure Time (s)	(C) Solid-Liquid Ratio	(D) Extraction Time (h)	(E) Extraction Temperature (°C)	Polysaccharide Yield (%)
1	1.2	60	1:40	3	80	13.16
2	1.2	60	1:40	4	90	13.46
3	1.2	75	1:50	3	80	9.19
4	1.2	75	1:50	4	90	10.34
5	1.4	60	1:50	3	90	13.01
6	1.4	60	1:50	4	80	12.81
7	1.4	75	1:40	3	90	13.71
8	1.4	75	1:40	4	80	15.63
K_1j_	46.16	52.45	55.96	49.08	50.78	
K_2j_	55.16	48.87	45.35	52.24	50.53	
k_1j_	11.54	13.11	13.99	12.27	12.70	
k_2j_	13.79	12.22	11.34	13.06	12.63	
R_j_	2.25	0.89	2.65	0.79	0.06	

Note. K_1j_ denotes the sum of the test results (Polysaccharide yield) corresponding to levels of 1 on any column, K_2j_ is the same. k_1j_ = K_1j_/S, where S is the number of occurrences of each level on any column, and K_2j_ is the same. R_j_ is the extreme variance, R_j_ = k_j_max − k_j_min. The smaller the extreme variance, the smaller the effect of the factor in that column on the experimental results.

**Table 4:** The results of comparison and verification of steam explosion (SE)-assisted extraction effects.

Samples	PEP	SEPEP
Polysaccharide yield (%)	7.52 ± 0.08	17.89 ± 0.40 ****

Note. Each value is represented as the mean ± SD (*n* = 3). “****” means statistically significant differences at *p* < 0.0001.

## Data Availability

The original contributions presented in the study are included in the article, further inquiries can be directed to the corresponding author.
